# Scale‐up/Scale‐down of microbial bioprocesses: a modern light on an old issue

**DOI:** 10.1111/1751-7915.12732

**Published:** 2017-05-29

**Authors:** Frank Delvigne, Henk Noorman

**Affiliations:** ^1^ TERRA Research CenterMicrobial Processes and Interactions (MiPI)University of LiègeLiègeBelgium; ^2^ DSM Biotechnology CenterDelftThe Netherlands; ^3^ Department of BiotechnologyTechnical University DelftDelftThe Netherlands

## Abstract

The bio‐economy is in transit from innovation to commercialization. The bioprocess industry is expected to increasingly deliver bio‐products to the market, in large amounts, at high quality and at competitive cost levels. This requires flawless start‐up of new large‐scale bioprocesses and continuous improvement of running processes. Fermentation scale‐up and operation can benefit from recent advances in three areas: 1. computation‐driven design of scale‐down simulators, 2. omics‐driven metabolic engineering and 3. sensing and understanding of population heterogeneity. Integration of these fields requires a unified computational approach, linked to big data and simulated reality frameworks, of which the contours are becoming visible today.

## Introduction

Today, scale‐up in the fermentation industry is still driven by physical guidelines (Delvigne *et al*., 2006) or heuristic approaches but does not consider individual cellular properties for *ab initio* and *in silico* design (Takors, 2012), nor high‐resolution data on fluid dynamics. Pioneering studies were performed by Lapin *et al*. (2006) but could not exploit the full predictive potential because details of metabolic and transcriptional regulatory responses were not yet known and computational capacities limiting. There thus remains an urgent need to pass the old scale‐up hurdles by the concerted application of modern systems and synthetic biology.

In a recent study, Takors and co‐workers described the applications of omics‐based diagnostics to bioprocess scale‐up (Simen *et al*., 2017). A two‐compartment scale‐down reactor (SDR), able to mimic glucose and ammonia fluctuations typically encountered in large‐scale bioreactors, has been used in order to investigate the transcriptional response of *E. coli*. The authors demonstrated that on the short term, the ppGpp‐dependent stringent response was induced when cells travelled the heterogeneous section of the SDR. On the long term, RNAseq analyses showed that around 400 genes were repeatedly switched on and off when crossing the two compartments of the SDR. This response induced an increased ATP demand of 15%. The analysis showed potential target genes associated with increased ATP demand for metabolic engineering. Such approach was previously used by the same research team for re‐designing an *E. coli* strain with increased glucose uptake capacity to cope with nutrient‐limiting conditions typically found in industrial fed‐batch bioprocess (Michalowski *et al*., 2017).

**Figure 1:**
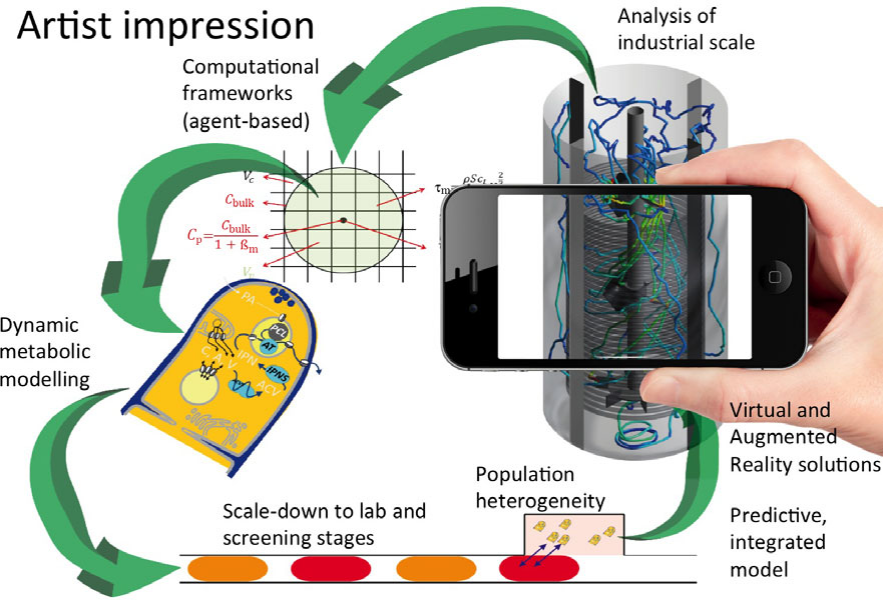
Overview of the scientific topics that have to be integrated in modern scale‐down studies and more generally for a better understanding of the impact of environmental fluctuations on microbial cell physiology in large bioreactors.

These new findings point out that predicting microbial physiological responses to environmental fluctuations remains a challenge but also that there are vast potential applications. This then is a good opportunity for presenting a modernized version of the scale‐down approach, i.e. an outlook on the different items that have to be considered for a better understanding of bioprocess scale‐up and the behaviour of cell populations under industrial process‐related conditions (Fig. 1).

The bioprocess industry more than ever needs new, efficient and sustainable routes to manufacture bio‐products. Bioprocesses use the power and versatility of nature via microorganisms that make the bio‐products from renewable feedstocks. These microorganisms today can be extensively re‐programmed into efficient cell factories. However, the gap between the cell environment at lab‐scale – where the engineering is performed – and production scale is still causing gross feedstock and asset utilization inefficiencies and is a barrier to fast and successful scale‐up. This bottleneck has become one of the most prominent risks for the bio‐economy, which is in transition from innovation (supported by the revolutions in metabolic engineering and molecular biology) to commercialization. Development and application of computational approaches to design better scale‐down simulators are expected to enable faster scale‐up and improve the energy and resource efficiency of biorefineries, which will accelerate the penetration of bio‐innovations to the markets. This bio‐based drive is essential to help solving the mega‐issues of climate change, food security and energy supply. The integrated computational solution proposed will further contribute to reconciliation of two main, competing, business success factors: speed and quality (Noorman and Heijnen, 2017).

In recent years, significant advances have been made at the level of the following three aspects:

- Scale‐down simulators like stirred tank/plug flow reactor set‐ups or devices for metabolic stimulation of the cells are already established and ready‐to‐use (Neubauer and Junne, 2010). Additionally, profound know‐how for performing computational fluid and reaction dynamics (CFD‐CRD) is provided linking hydrodynamics with cellular activities (Euler–Lagrange models) to simulate the impact of large‐scale heterogeneities (Delafosse *et al*., 2015; Haringa *et al*., 2017). Against this background, lifelines of cells in large‐scale fermenters can be reproduced to investigate their short‐ and long‐term interactions under real production conditions.
- The recent application of comprehensive scale‐down studies unravelled the massive metabolic and transcriptional response of cells in large‐scale bioreactors (Loffler *et al*., 2016; Simen *et al*., 2017). Such data provide a fruitful ground for uncovering key regulation phenomena and translating them in condensed models which can be used to predict and improve scale‐up performance. Additionally, metabolic engineering targets can be derived by identifying dominant transcriptional changes as a guideline for smart genome reduction.
- Smart synthetic bio‐sensors have been developed to link native regulatory responses with online detectable signals – or, even smarter – with fermentation control to screen and select for robust populations of choice. Such advances allows considering cell‐to‐cell heterogeneity among a population cultivated in large‐scale bioreactors (Delvigne *et al*., 2014).

All these aspects need to be linked in a unified computational approach for highest resolution and precision. From the computational approach to the much more powerful virtual reality and augmented reality solutions is only a small step and will further assist to overcome the challenges of the bioprocess industry. This step is actually rendered technically well‐feasible due to the recent advances made in big data analysis (i.e. cognitive software, machine learning and artificial intelligence that can be used for processing complex data; Schadt *et al*., 2010).

## Conflict of interest

None declared.
